# When combinatorial processing results in reconceptualization: toward a new approach of compositionality

**DOI:** 10.3389/fpsyg.2013.00677

**Published:** 2013-10-01

**Authors:** Petra B. Schumacher

**Affiliations:** Department of English and Linguistics, Independent Emmy Noether Research Group, Johannes Gutenberg University of MainzMainz, Germany

**Keywords:** ERP, metonymy, discourse updating, reference shift, late positivity, experimental pragmatics

## Abstract

Propositional content is often incomplete but comprehenders appear to adjust meaning and add unarticulated meaning constituents effortlessly. This happens at the propositional level (*The baby drank the bottle*) but also at the phrasal level (*the wooden turtle*). In two ERP experiments, combinatorial processing was investigated in container/content alternations and adjective-noun combination transforming an animate entity into a physical object. Experiment 1 revealed that container-for-content alternations (*The baby drank the bottle*) engendered a Late Positivity on the critical expression and on the subsequent segment, while content-for-container alternations (*Chris put the beer on the table*) did not exert extra costs. In Experiment 2, adjective-noun combinations (*the wooden turtle*) also evoked a Late Positivity on the critical noun. First, the Late Positivities are taken to reflect discourse updating demands resulting from reference shift from the original denotation to the contextually appropriate interpretation (e.g., the reconceptualization form animal to physical object). This shift is supported by the linguistic unavailability of the original meaning, exemplified by copredication tests. Second, the data reveal that meaning alternations differ qualitatively. Some alternations involve (cost-free) meaning selection, while others engender processing demands associated with reconceptualization. This dissociation thus calls for a new typology of metonymic shifts that centers around the status of the involved discourse referents.

## Introduction

One of the central questions in meaning composition involves the interpretation of utterances like (1)–(4). The sentence pairs have in common that the same expression stands for different things in the world (Dickens referring to the author vs. Dickens referring to a physical object/informational content; ham sandwich referring to something edible vs. the ham sandwich eater; etc.). How does the processing system arrive at different meanings and add unarticulated meaning aspects? What kind of information is consulted during these processes?

(1) a. The girl read Dickens.
b. The girl met Dickens.
(2) a. They protested during Vietnam.
b. They traveled around Vietnam.
(3) a. He put the beer on the table.
b. He brewed beer in the basement.
(4) a. The ham sandwich at table 2 wants to pay.
b. The ham sandwich is delicious.

Meaning extension or metonymy of this sort has been discussed as a semantic but also pragmatic phenomenon. Semantic approaches emphasize the contribution of lexical representation during composition (cf. e.g., Bierwisch, 1983; Copestake and Briscoe, 1995; Pustejovsky, 1995). On the one hand, the application of lexical derivational rules (such as producer-for-product, place-for-event or portioning) may generate different meanings. On the other hand, underspecified lexical representations may interact with context and conceptual structure to select a contextually relevant meaning. Pragmatic approaches in turn focus on speaker intention and conversational principles and argue that certain expressions are used to maximize informativity and efficiency (e.g., Nunberg, 1979; Horn, 1984; Nunberg and Zaenen, 1992; Blutner, 1998; Egg, 2004; Ward, 2004; Recanati, 2010).

From a processing perspective, experimental research has revealed different patterns for the examples presented above. A number of eye tracking studies have been conducted to assess the comprehension of cases like (1) and (2) reporting no differences between the (a)- and b)-cases (Frisson and Pickering, 1999, 2001; Frisson, 2009). These findings have been taken as evidence for underspecified representations. By contrast, the application of a portioning rule as required for (3) or a grinding rule (“There was chicken in the soup.”) during mass/count alternations has evoked different eye movement profiles for the “derived” meaning (Frisson and Frazier, 2005). These data suggest that the application of a lexical derivational rule exerts processing demands. Furthermore, grinding showed additional later effects, possibly indicating that meaning shift involving individuation (in the portioning case) may be conceptually easier.

What can we make of these differences in the eye tracking measures? They suggest that not all instances of metonymy should be treated alike. Mass/count alternations are more costly than producer/product and place/event alternations, which might reflect the application of a lexical rule in the former case and a different compositional operation in the latter cases. Two further observations point toward a complex and multifaceted system of meaning composition. Traditionally, polysemous expressions have been compared with homonyms (i.e., expressions with different meanings that are not related conceptually like *bank*). Processing homonymy—but not polysemy—is sensitive to the number of available meanings for a given expression and frequency of occurrence (Rayner and Duffy, 1986; Frazier and Rayner, 1990; Frisson and Pickering, 1999). This was taken as an indication that polysemous expressions are not committed to a particular or basic meaning but come with underspecified representations. Second, while producer/product alternations engender no processing costs, complement coercion (“The student began the book.”) shows extra processing demands (McElree et al., 2006a). Complement coercion (also known as logical metonymy) has been investigated extensively as an instance of enriched interpretation, where the predicate (*begin*) requires an event-type complement, which induces enriched composition when an entity-denoting expression like *book* is encountered. The computation of unarticulated event information (such as *reading the book*) registered processing costs across numerous experimental paradigms (McElree et al., 2001, 2006a; Pylkkanen and McElree, 2007; Kuperberg et al., 2010). This supports the view that different compositional operations are available to the language system to resolve not explicitly articulated meaning constituents.

Finally, ERP studies with cases like (4) registered enhanced processing costs for the shifted use (Schumacher, 2011, 2013). These cases differ from (1)–(3) in that a salient property is used to refer to an individual. When uttered in the context of a restaurant, *the ham sandwich* can be interpreted as “an individual contextually associated with the ham sandwich” (Nunberg, 1979; Lakoff and Johnson, 1980; Jackendoff, 1997). These meaning shifts are less systematic and conceptually integrated than the alternations exemplified in (1)–(3), suggesting that the underlying processes may be different requiring extra-lexical conceptual and inferential computation. The observed ERP differences were reflected in a Late Positivity for the shift in (4) (Schumacher, 2011, 2013). In turn, N400 differences were reported for complement coercion (Kuperberg et al., 2010) also pointing toward qualitatively different mechanisms. We will return to the implications of these differences below.

The processing data thus suggest that different operations guide combinatorial processing. In all of the cases sketched above, the type presupposition^1^ of the predicate is not met by the type of the argument and must be accommodated^2^; *read* requires a physical object, yet encounters a person, *pay* demands a person, yet encounters a physical object, etc.; for type compositional accounts see for instance Copestake and Briscoe (1995), Pustejovsky (1995), and Asher (2011). Type accommodation may be achieved by specification of underspecified representations or by application of a lexical derivational rule, and it may also have discourse-pragmatic consequences such that meaning shift results in reconceptualization and the establishment of a new discourse entity. This latter effect can be illustrated by copredication and coordination tests that show that access to the unshifted meaning is blocked in certain cases like (5a) (cf. Cruse, 1986; Copestake and Briscoe, 1995). This crucially suggests that the discourse representation of (5) is changed and reconceptualized from the physical object to a person-denoting referent, while in (6) both types (the person and the object·information) are accessible.

(5) a. ^*^The ham sandwich at table 2 paid and was stale.
b. The ham sandwich at table 2 paid and went home.
(6) a. Tim's grandmother had read Dickens before she met him at a Christmas party.
b. Tim's grandmother had read Dickens before she placed it on the shelf.

This test is not unproblematic because it can interact with other linguistic factors. Cases that allow for copredication are a good indication of a single representation associated with the different instantiations, but negative evidence in turn cannot immediately be taken as evidence for reference transfer and should be taken with a grain of salt, since other criteria that are independent of the meaning shift may be responsible for the failed copredication or coordination such as coherence, morphosyntax or the combination of incompatible predicates (cf. Cruse, 1986; Copestake and Briscoe, 1995). In (6b) for instance, the morphosyntactic gender mismatch between the anaphor and the antecedent could add an unwanted negative flavor to the object-denoting example^3^. However, such negative evidence would be caused by morphosyntactic mismatches. In German, where the author and his work may share the same gender feature, copredication yields an acceptable outcome. Imagine a student skimming over a journal paper (*der Aufsatz*, “the paper”- masculine) by Kant:

(7) a. Nachdem Anne Kant überflogen hatte, kopierte sie ihn. after Anne Kant skim-over had copied she him “After Anne had skimmed over Kant, she copied it.”

It is therefore important to construct copredication tests with great care and to treat the results cautiously.

In the following, I want to demonstrate that these differences associated with the referential instantiation of an expression are also reflected in the underlying processes. Accordingly, application of a lexical rule or contextual specialization should be functionally differentiated from discourse-internal restructuring and reconceptualization. The ingredients for the underlying language architecture are a rich lexical representation and mechanisms for the adjustment of meaning that satisfy type presuppositions as well as principles of discourse representation (cf. e.g., Pustejovsky, 1995; Jackendoff, 1997; Asher, 2011). The processing system makes available different mechanisms leading to meaning adjustment (cf. e.g., underspecification vs. rule application or coercion, co-composition, selective binding in Pustejvosky's framework), but the critical distinction between different kinds of meaning shift that I want to highlight here is between entities that require updating of discourse representation structure and reconceptualization (“referent shift”) and meaning shift that involves a type shift or meaning adjustment without altering the respective discourse referent in fundamental ways. The underlying processes can thus be attributed to discourse operations or semantic operations. The Late Positivity observed for *ham sandwich* alternations may hence be credited to reconceptualization and referent shift and should not be evoked by meaning alternations that simultaneously maintain multiple meanings.

Electrophysiological measures have not been used systematically for the investigation of unarticulated meaning constituents so far. At the word level, related work has investigated the processing of semantically “rich” lexical representations with a focus on the density of semantic associations and shared features. Following this line of research, Taler and colleagues assessed the contribution of multiple meanings to online processing. Presenting word lists in a lexical decision task, they found more pronounced N400-modulations for homonymy (e.g., *bank* representing a financial institution or side of the river) than for metonymy (e.g., *chicken* allowing for an animal or meat reading) (Taler et al., 2009). The N400-amplitude is also inversely related to the number of meanings (Taler et al., 2013). This indicates that the amount of related interpretations facilitates lexical decisions and points toward fast access to rich lexical representations. In complement coercion (“The journalist began/wrote the article”), an N400 was observed when an entity-denoting expression had to be enriched toward an event reading (Baggio et al., 2010,^4^; Kuperberg et al., 2010). The relevant event information may be retrieved from the qualia structure of the nominal expression [e.g., *article*: telic-role: *read*(e, x, y); agentive-role: *write*(e, x, y)] in interaction with world knowledge (cf. Pustejovsky, 1995; Jackendoff, 1997; Lascarides and Copestake, 1998). For effects of the different qualia roles and context see Verspoor (1997) and Lapata et al. (2003). This lexical operation may be costly (as suggested by the pronounced N400), but it crucially does not change the referent's discourse representation (*article*).

Ever since its discovery as a language-related neural response, the nature of the N400 has been approached from various angles and many factors have been identified that influence the N400 during word, sentence, and text level comprehension. The negativity has been associated with processes of semantic activation, retrieval, integration, and expectation (see Kutas and Federmeier, 2011 for a summary of thirty years of research). The N400 to complement coercion could for instance be viewed in terms of retrieving relevant qualia information (as outlined above) but it could also reflect the more basic operation of detecting the semantic type mismatch [as suggested by Kuperberg et al. (2010) who (1) show a similar N400 for animacy violations and (2) report an additional sentence-final anterior positivity for complement coercion which they attribute to reanalysis and retrieval].

A Late Positivity peaking around 600 ms after the onset of a critical expression has also been reported to be reflective of compositional operations. Early ERP research has found late positive deflections for morphosyntactic violations (e.g., Osterhout and Holcomb, 1992; Hagoort et al., 1993; Harris et al., 2000), but this narrow functional characterization has been challenged by more recent findings of late positive effects that lack a syntactic explanation. Late positive effects have for instance been associated with semantic reversal anomalies (for an overview see Bornkessel-Schlesewsky and Schlesewsky, 2008) but also with referent shift as exemplified by (4) above (Schumacher, 2011, 2013). The positivity engendered by semantic reversal anomalies has been attributed to conflict resolution mechanisms triggered by implausibility or violations of selectional restrictions (cf. e.g., Van Herten et al., 2005; Kuperberg et al., 2007; Bornkessel-Schlesewsky and Schlesewsky, 2008; Paczynski and Kuperberg, 2011). Utterances such as “The fox that hunted the poacher stalked through the woods” induce for instance a mismatch between the interpretation arrived at on the basis of semantic and syntactic analysis and world knowledge. The detection of this mismatch and possibly its resolution exerts processing demands. In the referent shift case in (4), the utterance only makes sense if one construes an interpretation where *the ham sandwich* refers to a person associated with it, such as *the ham sandwich eater.* Here again the qualia structure of *ham sandwich* may provide the relevant information for the intended meaning [telic-role: *eat*(e, x, y)]. Yet this information is utilized to transfer reference from the physical object [*ham sandwich*(y)] to the agent of the telic event [i.e., the *eater*(x)] as suggested by the copredication test illustrated by (5). An account of the Late Positivity that considers the modification of the referential representation is a natural extension of independent findings from referential processing where the Late Positivity has already been associated with discourse updating in cases where new referents are introduced or discourse structure must be revised (Burkhardt, 2006, 2007; Brouwer et al., 2012; Wang and Schumacher, 2013).

Finally, not only eye-tracking studies have registered late effects during compositional processing but a few ERP studies have also reported processing costs on subsequent words in the sentence. Kuperberg et al. (2010) show a positive deflection at the word following the mismatching noun and a sustained negativity further downstream for an animacy violation condition (“The journalist astonished the article”). They also report sentence-final positivities for animacy violation and complement coercion. Another study found a sustained negativity or possibly a sequence of N400 effects for complement coercion relative to the noun (Baggio et al., 2010). Furthermore, meaning adjustments involving enrichment of an accomplishment (“The girl was writing a letter when her friend spilled coffee on the paper.”) yielded a sustained anterior negativity at the verb following the noun that induced the accomplishment reading^5^ (Baggio et al., 2008). A sustained negativity has also been associated with working memory demands when events are not processed in their chronological order (Münte et al., 1998). These prolonged or downstream effects have thus been suggested to reflect ongoing semantic and discourse-based computation.

The current investigation aims at testing the hypothesis that meaning adjustment resulting in reconceptualization and referent shift evokes distinct processing patterns. This is achieved by looking at different sorts of predication. Verb-induced meaning adjustment (Experiment 1) presents an investigation of content/container alternations induced by verb-inherent requirements. Adjective-induced meaning adjustment (Experiment 2) extends the hypothesis to the phrasal domain where adjective-noun combinations are at stake. The overarching questions are what are the underlying neural mechanisms during combinatorial processing and what do they tell us about possible typologies of meaning adjustment?

## Verb-induced meaning adjustment (experiment 1)

Since shifts involving *ham sandwich*-style expressions could be considered relatively exceptional cases of meaning adjustment, it is important to investigate the compositional processes underlying more systematic meaning alternations. We use container/content alternations and investigate meaning shifts in both directions. In (8) *bottle* is used to refer to its content, in (9) *beer* refers to a contextually appropriate container. In both cases, the shift is enforced by a clash between the type presupposition of the predicate and the default type of the argument [illustrated in (b)]. In the container-for-content alternation, the required type may be recruited from the qualia structure of *bottle*, which offers functional information (bottles as containers hold liquids and other stuff) (8.c). The specification of the actual content appears to be relatively vague and highly context-dependent (in the song “Johnny drank the bottle,” the containable most likely represents beer or whiskey; if we know that Johnny is a five-month-old, milk is a more likely option).

The content-for-container usage is also known as mass-count alternation, which can be derived on the basis of the portioning rule (Copestake and Briscoe, 1995). This lexical rule takes as input a mass noun and returns a count noun. There are certain relations between ontological types—such as liquids can be contained in physical objects (9.c)—whose specification is determined by encyclopedic knowledge, and these relations are made available during compositional processing to induce a meaning shift (cf. Copestake and Briscoe, 1995; Dölling, 1995) (9.d).

(8) a. Johnny drank the bottle^6^.
b. *drink*(e, x, y) & person(x) & liquid(y) ↔ *bottle*(y), physical object(y)
c. telic role (y): λy. bottle(y) & telic = λz λe'*. contain*(e', y, z) …
d. λy λx λe. *drink*(e, x, y) & *Johnny*(x) & *bottle*(y) & telic = λz λe'*. contain*(e', y, z)
(9) a. He put down the beer.
b. *put-down*(e, x, y) & person(x) & physical object(y) ↔ *beer*(y), liquid(y)
c. portioning rule R: **∀**y. liquid(y) → **∃**z. physical object(z) & CONTAINER(z, y)
d. λy λx λe. *put-down*(e, x, y) & *he*(x) & *beer*(y) & **∃**z. physical object(z) & CONTAINER(z, y)

The two meaning shifts may differ in a subtle way in that content-for-container relies on the application of a general lexical derivation rule, while container-for-content uses a variable (z) from the expression's qualia structure to satisfy type presuppositions. They also differ in that the former but not the latter allow copredication (10/11).

(10) a. Peter put down the beer and drank it a few minutes later.
b. Peter put down the beer and incidentally knocked it over a few minutes later.
(11) a. #Tim drank the bottle and dropped it.
b. Tim drank the bottle and chocked on it.

This suggests that both meanings remain accessible in the case of portioning (10). By contrast, the container meaning is blocked once the variable representing the containable has been established as referent (11). This moves the container-for-content alternation in the immediate vicinity of the reference shift observed for the *ham sandwich* cases. Whether these differences in referential instantiation have consequences for language processing is examined in Experiment 1.

### Methods

#### Participants

Twenty-two monolingual, native speakers of German participated in the reading experiment after giving informed consent (12 women; mean age: 22.36 years; range: 19–32 years). They were right handed (as assessed by a version of the Edinburgh handedness inventory Oldfield, 1971) and had normal or corrected-to-normal visual acuity. Two additional participants were excluded from the analysis due to excessive ocular artifacts.

#### Materials

Eighty pairs of items were created for container-for-content and content-for-container metonymy and their corresponding controls (cf. Table 1 and the supplemental data for a complete list of experimental items). To allow us to measure processing cost immediately at the noun, we utilized question-answer pairs that asked for the critical item thereby generating a high expectation for a container or content-denoting noun respectively in the target sentence (e.g., “What did Heinz drink hastily? - He drank the goblet hastily.”). For a similar approach see Schumacher (2011, 2013) on non-systematic metonymy.

**Table 1:** **Sample stimuli for Experiment 1**.

**CONTAINER-FOR-CONTENT**	
(A.1) Meaning shift	Was | hat | Heinz | hastig | getrunken?
	what has Heinz hastily drunk
	“What did Heinz drink hastily?”
	Er | hat | **den Becher** | hastig | getrunken.
	he has the goblet hastily drunk
	“He drank **the goblet** hastily.”
(A.2) Control	Was | hat | Rolf | wie seinen Augapfel |gehütet?
	what has Rolf like his eyeball guarded
	“What did Rolf guard jealously?”
	Er | hat | **den Becher** | wie seinen Augapfel | gehütet.
	He has the goblet like his eyeball guarded
	“He guarded **the goblet** jealously.”
**CONTENT-FOR-CONTAINER**	
(B.1) Meaning shift	Was | hat | Asterix | an seinem Gürtel | festgeschnallt?
	what has Asterix to his belt fastened
	“What did Asterix fasten to his belt?”
	Er | hat | **den Zaubertrank** | an seinem Gürtel | festgeschnallt.
	he has the magic potion to his belt fastened
	“He fastened **the magic potion** to his belt.”
(B.2) Control	Was | hat | Miraculix | vor dem Eintreffen | der Römer | gebraut?
	what has Miraculix before the arrival the Romans brewed
	“What did Miraculix brew before the arrival of the Romans?”
	Er | hat | **den Zaubertrank** | vor dem Eintreffen | der Römer | gebraut.
	he has the magic potion before the arrival the Romans brewed
	“He brewed **(the) magic potion** before the arrival of the Romans.”

Critical expressions are marked in bold.

All items were checked for their plausibility by means of a paper-and-pencil plausibility test with a group of participants who were not recruited for the electroencephalogram (EEG) experiment later on as well as a post-test with the participants in the EEG experiment. All critical items were distributed across eight lists and interspersed with 15 plausible fillers and 15 implausible fillers. Participants were asked to rate the plausibility of the question-answer pairs on a 5-point scale (1: plausible, 5: implausible). In the pre-test, 52 students from the University of Cologne filled out the questionnaires (46 women; 19–38 years old; mean age: 25.7). In the post-EEG test, data from 22 participants (detailed above) were analyzed. The analyses revealed no differences between the meaning shift and the control condition in the content-for-container pairs (all *F*s < 1.4), but a reliably lower plausibility rating for the container-for-content expressions relative to their controls [pre-test: *F*_(1, 39)_ = 38.85, *p* < 0.001; post-test: *F*_(1, 39)_ = 19.42, *p* < 0.001]. Importantly, the responses to the critical items yielded overall more plausible ratings than the implausible fillers. See Table 2 for mean ratings.

**Table 2:** **Mean plausibility ratings for items from Experiment 1; standard deviations in parentheses (1, plausible; 5, implausible)**.

	**Pre-test**		**Post-test**	
	**Meaning shift**	**Control**	**Meaning shift**	**Control**
Container-for-content	2.67 (1.04)	1.40 (0.57)	2.10 (0.92)	1.27 (0.033)
Content-for-container	1.34 (0.53)	1.31 (0.49)	1.32 (0.46)	1.22 (0.28)
Implausible filler	4.77 (0.56)		4.92 (0.22)	

The container-for-content metonymies included an additional exploratory factor “modification of the head noun” where half the items were specified for their content (e.g., *water bottle, tea kettle, pasta pot*) and the other half was not (e.g., *bottle, kettle, pot*). This factor was included to test whether the availability of content-denoting information facilitates meaning adjustment. The plausibility ratings revealed no effect of modification although the content-modifying expressions registered slightly better ratings. The factor “modification” also entered the ERP analysis but yielded no reliable effects. Because this was just an exploratory factor and for reasons of readability, this factor is no longer mentioned in the following sections.

For the ERP study, the critical items were distributed across two lists. An additional 120 question-answer pairs were included as fillers yielding a total of 280 items presented to each participant in a pseudo-randomized manner. To incite participants to pay attention to the stimuli, they were asked to perform a word recognition task after each item. Participants performed this task correctly in more than 95% of the trials.

#### Procedure

The experiment was carried out in a dimly lit, sound-proof booth. Participants were seated comfortably in front of a computer monitor and were instructed to read sentences for comprehension in rapid serial presentation modality and respond to a probe recognition task after each trial.

Trials were presented visually in the center of the monitor in yellow letters on a blue background. Following the presentation of an asterisk for 400 ms, each stimulus was presented in segments (marked by vertical bars in the sample items in Table 1) with single words occurring for 400 ms, two-word segments for 450 ms and three-word segments for 500 ms with an inter-stimulus interval (ISI) of 150 ms. Each trial ended with a blank screen of 500 ms and the presentation of 3 question marks for 500 ms followed by a word probe for the recognition task, which stayed on the screen until the participant responded but no longer than 3000 ms. After an intertrial interval of 1000 ms, the next trial was presented.

The probe recognition task required participants to press one of two buttons on a game pad, indicating whether the presented word occurred in the trial or not. Yes and no responses were evenly distributed across all items and blocks. The assignment of the left and right buttons was counterbalanced across participants.

Following electrode application, each participant performed a short practice session before the experiment was administered in seven blocks with short breaks in-between. Each block consisted of 40 trials.

#### EEG recording

The EEG was recorded from 26 Ag/AgCl scalp sites according to the international 10–20 systems of electrode placement (ground: AFZ), which were mounted in an elastic cap (*Easycap*, Munich, Germany). All active channels were referenced to the right mastoid online and rereferenced offline to averaged mastoids. The electrooculogram (EOG) was recorded from four electrodes placed around the participant's eye to control for artifacts due to horizontal and vertical eye movements. Electrode impedances were kept below 5 kΩ during EEG recording. All EEG and EOG channels were amplified using *BrainAmp* (Munich, Germany) and digitized at a rate of 500 Hz.

#### Data analysis

Before averaging, EEG data were processed for ocular artifacts (automatic EOG rejection criterion: ±40 μ V) and bandpass filtered with 0.3–20 Hz offline to exclude slow signal drifts. ERPs were time-locked to the onset of the critical word and averaged per condition and participant. Trials that contained artifacts or registered an incorrect or timed-out response in the recognition task (12.1%) were excluded from averaging. The analysis was carried out separately for lateral and midline sites. The lateral electrodes were grouped by topographical region of interest (ROIs) which entered the analysis as additional factor with four levels: left-anterior (F3, F7, FC1, FC5), left-posterior (CP1, CP5, P3, P7), right-anterior (F4, F8, FC2, FC6), right-posterior (CP2, CP6, P4, P8). The midline analysis (MID) included the six midline sites as levels (Fz, FCz, Cz, CPz, Pz, POz). Repeated measures ANOVAs were performed separately for the two types of metonymies due to inherent differences in the material. Each analysis included the factor CONDITION (Meaning Shift vs. Literal) and ROI/MID and was carried out on the mean amplitude value per condition in time windows determined by visual inspection. Statistical analyses were performed on a time interval from the onset of the critical expression till 2000 ms thereafter to test for potential late ERP effects. All analyses were carried out hierarchically so that only significant interactions (*p* < 0.05) were resolved. Huynh–Feldt adjustment was applied when the analysis involved factors with more than one degree of freedom in the numerator (Huynh and Feldt, 1970).

### Results

#### Container-for-content

**Figure 1:**
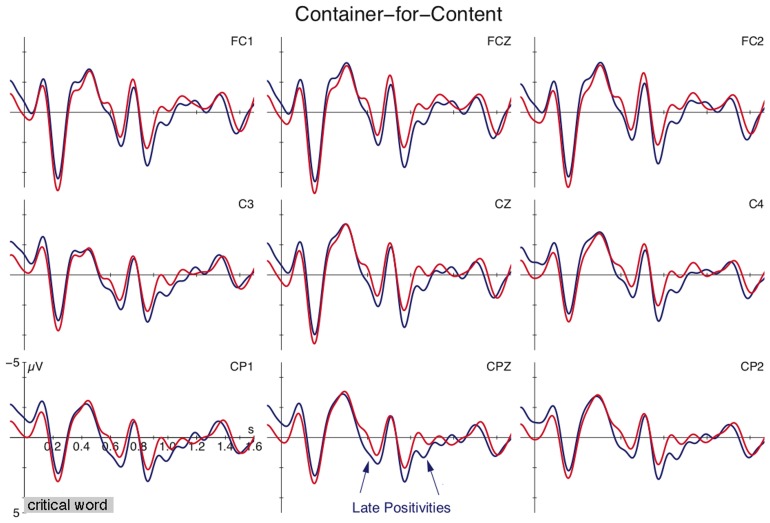
**Grand average ERPs time-locked to the critical container expression (*den Becher*, “the goblet”) at selected electrodes for the meaning shift (blue) and control condition (red)**. The onset of the critical expression is at vertical bar; the presentation of the subsequent segment started at 0.6 s. Negativity is plotted upwards.

Figure 1 shows the grand-average ERPs for container expressions (e.g., *goblet, kettle*) used to refer to containers or their content relative to the onset of the critical expression (onset at vertical bar). The figure reveals a more pronounced positive deflection for the metonymic use over the literal use between 550–750 ms and between 900–1100 ms. This was confirmed by the statistical analysis which revealed a main effect of CONDITION in the time window between 550–750 ms [lateral analysis: *F*_(1, 21)_ = 12.98, *p* < 0.002; midline analysis: *F*_(1, 21)_ = 13.17, *p* < 0.002] and between 900–1100 ms [lateral analysis: *F*_(1, 21)_ = 21.66, *p* < 0.001; midline analysis: *F*_(1, 21)_ = 13.34, *p* < 0.002]. Additional tests in the window between 350–500 ms registered no reliable effect (*F* < 1).

We performed an additional correlation analysis on the offline plausibility ratings and mean ERP difference scores, since Late Positivity effects have been associated with implausibility. One outlier (with a mean plausibility score of 4.6) was excluded from the analysis. The correlations did not reach significance (550–750 ms: Pearson's *r* = −0.003, *p* = 0.98; 900–1100 ms: *r* = −0.18, *p* = 0.29).

#### Content-for-container

**Figure 2:**
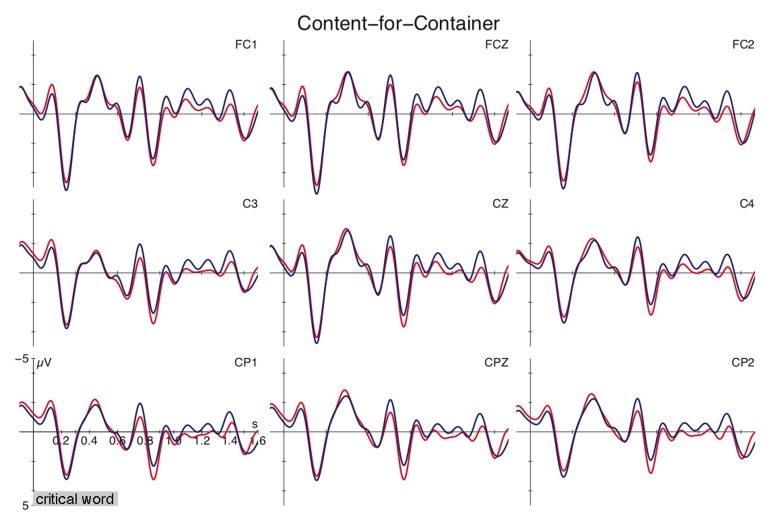
**Grand average ERPs time-locked to the critical content expression (*den Zaubertrank*, “the magic potion”) at selected electrodes for the meaning shift (blue) and control condition (red)**. Time ranges from 100 ms before till 1600 ms after the onset of the critical expression (onset at vertical bar).

The grand-average ERPs relative to the onset of content expressions (e.g., *magic potion*, *beer*) used to refer to the content or an associated container are depicted in Figure 2. No differences between the two conditions are observable in the figure. Statistical analyses support this for the time-windows between 350–500 ms as well as between 550–750 ms and 900–1100 ms [all *F*s < 1]. Additional 100 ms analyses over the entire interval registered no reliable effects.

### Discussion

Experiment 1 investigated two meaning alternations associated with containers and containables. In a generative lexical approach, these two alternations are couched within rich lexical representations making available a general rule like portioning or allowing for interactions between the lexical representations of the predicate and its argument, in the current case qualia structure. From a discourse-dynamic perspective, container-for-content alternations stand out by shifting their referent, which is evidenced by copredication tests, while content-for-container alternations afford access to both meanings. A provisional possible solution detailing the specifics of the lexical representations for this contrast is sketched in the following. A derivational rule like the portioning rule may be freely available during combinatorial processing. Once added to the representation [e.g., (9d): λy λx λe. *put-down*(e, x, y) & *he*(x) & *beer*(y) & **∃**z. physical object(z) & CONTAINER(z, y)] the relation between container (z) and containable (y) is instantiated and both variables are equally accessible and switching between the two readings is free of cost. This close connection may be due to the natural ontological relation between substances and their respective containers. In contrast, the container-for-content alternation builds on information from the container's qualia structure, in particular the functional information specifying the purpose of the object [e.g., (8c): telic role (y): λy. bottle(y) & telic = λz λe'. *contain*(e', y, z)]. In this case, the content is a variable (z) in the telic quale, which has to be extracted and promoted to independent discourse representational status, resulting in referential shift.

The current ERP data support a dissociation of these two alternations. Content-for-container alternation shows no differences in the processing profiles of content and container usages (cf. Figure 2). In turn, container-for-content alternation exerts additional computational demands when referring to the content reading. This extra cost is reflected in a sequence of positivities between 550–750 ms and 900–1100 ms after the onset of the critical expression (cf. Figure 1). The application of the portioning rule does not exert costs reflected in ERP differences, suggesting that the two meanings represent two sides of the same coin. The content and its corresponding packaging appear to be directly connected with each other when using the mass term. This correspondence does not come entirely for free as suggested by reading time differences reported in previous research [longer gaze duration in Frisson and Frazier (2005)]. But the underlying operations appear to be fundamentally different from the container-for-content alternation. The Late Positivities for the latter alternation are taken to reflect discourse updating and reconceptualization processes. This is substantiated by previous positivities in discourse processing and by effects in the copredication test as outlined above^7^.

First, the data conform to the reference shifting account for the *ham sandwich* alternations. Second, diagnostics such as copredication, coordination, and directionality may shed light on the discourse nature of the corresponding referents (Cruse, 1986). Effects of copredication have been used before to illustrate differences in terms of type shifting (where copredication fails) and meaning selection (where different meanings are maintained) (Godard and Jayez, 1993; Copestake and Briscoe, 1995). As pointed out in the introduction, positive evidence is a more reliable source since other factors may account for failed copredication or coordination (cf. Cruse, 1986; Copestake and Briscoe, 1995). Crucially, such factors do not seem to apply to the example in (11). But these effects should also be tested in German, since language-specific repercussions are possible. Here (12) yields the concordant effects, while the test is somewhat more tricky with container-for-content expressions and imposes independent constraints of agreement and coherence. Yet, modifying a container-for-content expression like *glass* with a container-describing attribute as in (13.a) appears to be less acceptable than the combination with content description (13.b). Furthermore, coordination of both meanings as in (13.c) is not possible.

(12) a. Peter stellte das Bier hin und trank es einige Minuten später.
  “Peter put down the beer and drank it a few minutes later.”
b. Peter stellte das Bier hin und warf es einige Minuten später versehentlich um.
  “Peter put down the beer and accidentally knocked it over a few minutes later.”
(13) a. ?Tim trank noch ein Glas, das mundgeblasen war.
  “Tim drank yet another glass that was mouthblown.”
b. Tim trank noch ein Glas, weil es so schön prickelte.
  “Tim drank yet another glass because it sparkled so nicely.”
c. #Tim trank das mundgeblasene und prickelnde Glas.
  “Tim drank the mouthblown and sparkling glass.”

A new finding has been the additional positivity for container-for-content alternation on the segment following the critical expression. The positivity between 900–1100 ms resembles the one between 550–750 ms in terms of the broad topographical distribution. The extra positivity might be a spill-over effect reflecting enhanced computational costs in a case of meaning adjustment that is conceptually demanding because extra inferential reasoning might be needed to determine the intended content of the container (see also Frisson and Frazier, 2005 for a similar account of distinct conceptual demands for grinding over portioning). In this case, both positivities could be taken to reflect reconceptualization, where the second positivity echos the extra demands associated with this particular type of metonymy. Alternatively, the initial positivity could reflect the mismatch detection. Since late positivities have also been associated with well-formedness and plausibility, such an account appears to be reasonable as well. The second positivity might then reflect the attempt to rescue the interpretation via meaning adjustment. Such a two-step reconceptualization appears surprising given that other instances of meaning adjustment have not shown this pattern (e.g., *ham sandwich* alternation). Yet, it could again be argued that container-for-content alternations are conceptually more demanding.

A tentative way to choose between these two explanations is to compare the ERP responses with the offline plausibility data. Correlation analyses between the mean ERP difference score of the positivities and offline plausibility ratings were not significant. In particular, the offline ratings had no bearing on the initial positivity (Pearson's *r* = −0.003, *p* = 0.98), which according to the error detection account should at least show a trend. The absence of a correlation then weakens the plausibility account.

In sum, the data from Experiment 1 provide evidence for two distinct neural operations, one involving meaning selection within the lexicon, which appears to be relatively inexpensive during combinatorial processing, the other requiring extra computational demands, possibly associated with the modification of a discourse referent. The copredication test supports this view, but the container-for-content judgments are not as clear as the ham sandwich judgments. To strengthen the current claim, we turn to a different type of composition involving adjective-noun pairs, which also require modification and reconceptualization of a discourse referent. By looking at a new type of meaning adjustment, the findings from Experiment 1 can be evaluated against a broader background. It further provides added value to the current debate in extending investigations of meaning adjustment to the phrasal domain.

## Adjective-induced meaning adjustment (Experiment 2)

Previous research has started to investigate the comprehension of adjectival predicates in which an event reading must be obtained during the combination of adjectives and entity-denoting nouns [cf. “The climber imagined the ice survivable” in McElree et al. (2006b); “the quick route” in Frisson et al. (2011)]. In both cases, processing costs were observed in response to the type conflict. Within typed feature structures, the type adjective has subtypes that specify specific qualia roles (Copestake and Briscoe, 1995). Accordingly, *quick* is a telic-adjective that combines with an event (e.g., *quick start*) or that selects the telic role of an entity to specify its purpose and function (*quick route*).

Similarly, material adjectives select for an inanimate entity that can be made up of a particular material (*wooden trunk*). But how do we arrive at the interpretation of *wooden turtle*? The nominal expression must be coerced from an animal denotation to a physical object representation of a turtle. Crucially, simple combinatorial processing involving an intersective adjective and a nominal as in (14.b) does not yield the desired reading “object in the form of a turtle made of wood.” Due to the material clash (induced by animate vs. inanimate features), the combinatorial system must generate a selective interpretation with the formal quale of the nominal expression, which enumerates information about shape, color, magnitude, etc. (14.c). Alternatively, a derivational rule may be applicable to interpret a nominal expression as a visual representation (as suggested for pictures, statues, and the like) (Jackendoff, 1997) (14.d). Asher (2011) considers these combinations as instances of loose talk: the predication relation is loosened in such a way that “wooden turtles” are no longer turtles but something in the shape of a turtle (i.e., a contextually salient property). In any case, such an operation yields a transformation from the animal-denoting entity to the physical object. The adjective thus alters the type of the denotation (cf. also Kamp and Partee, 1995). This is also evidenced by the test in (15) showing that access to the original meaning of *turtle* is blocked. Based on the provisional account given above, copredication failure predicts that the underlying mechanism relies on qualia information (14.c) rather than a lexical rule that makes available both denotation (14.d).

(14) a. wooden turtle
b. λx. *turtle*(x) & *wooden*(x)
c. formal quale (x): λx. *turtle*(x) & shape(turtleshape, x) & color(greenish, x) & …
d. visual representation rule R: **∀**x. animate entity(x) → **∃**y. physical object(y) & VISUAL-REPRESENTATION-OF(y, x)
(15) a. #Die hölzerne Schildkröte verschlang das Salatblatt.
  “The wooden turtle gobbled the lettuce leaf.”

Whether this reconceptualization results in costs similar to the *ham sandwich* and container-for-content alternations is examined in Experiment 2. One potential caveat is related to the animacy mismatch induced by the adjective-noun combination. Animacy mismatches have previously engendered a biphasic N400—Positivity pattern (“The journalist astonished the article”; Kuperberg et al., 2010). The N400 was associated with featural mismatch detection, the positivity with semantic implausibility and reanalysis. This also connects to semantic reversal anomalies (cf. e.g., Van Herten et al., 2005; Kuperberg et al., 2007; Bornkessel-Schlesewsky and Schlesewsky, 2008) and to animacy effects in the computation of argument relations where animacy represents one of the features during prominence computation and has shown intricate cross-linguistic differences with varying mono- and biphasic patterns (Frisch and Schlesewsky, 2001; Bornkessel-Schlesewsky et al., 2011). Note however that while animacy figures prominently in *ham sandwich* alternations, this is not the case in container-for-content alternations, suggesting that the animacy mismatches are not the sole source of the Late Positivity (see also Paczynski and Kuperberg, 2011 for non-animacy related selectional restriction violations). But animacy effects may also arise as early as on the first constituent of an utterance showing a disadvantage (N400) for inanimate over animate entities (Weckerly and Kutas, 1999). Crucially, since the mismatch in *wooden turtle* is induced by an animate entity, the disadvantage for the inanimate feature goes against the current prediction.

### Methods

#### Participants

Thirty-three monolingually raised speakers of German participated in the experiment after giving informed consent (14 men; mean age: 22.8 years; range: 18–32 years). None of them had participated in Experiment 1.

#### Materials and procedure

Thirty pairs of items were constructed combining a material adjective (e.g., *wooden, iron, metallic*) with either a physical object (control condition) or an animate entity. See Table 3 for sample stimuli and the supplemental data for a full list of adjective-noun pairs. The critical nouns were matched for syllable length [mean: 2, standard deviation (*SD*): 0.5], and controlled for frequency of occurrence (http://wortschatz.uni-leipzig.de; mean-animate: 14.6, *SD*: 2.2, mean-inanimate: 14.0, *SD*: 2.2, *F* < 1) as well as gender marking across items.

**Table 3:** **Sample stimuli for Experiment 2**.

**Material adjective + noun**	
(C.1) Meaning shift	Die |hölzerne | **Taube** |befand |sich |auf |dem |Tisch.
	the wooden dove was-located REFL on the table
	“The wooden **dove** was on the table.”
(C.2) Control	Die |hölzerne | **Truhe** |befand |sich |neben |dem |Bett.
	the wooden trunk was-located REFL next-to the bed
	“The wooden **trunk** was next to the bed.”

Critical words are marked in bold.

Since animacy might have an effect above and beyond meaning adjustment, we also tested adjective-noun combinations that involve simple intersective adjectives (e.g., denoting color: *gray dove* vs. *gray shirt* or weight: *light cat* vs. *light crown*). We constructed thirty pairs of stimuli with the nouns of each pair matched for number of letters (mean: 5.2, *SD*: 1.2), number of syllables (mean: 1.8, *SD*: 0.6) and frequency (http://wortschatz.uni-leipzig.de; mean: 12.5, *SD*: 2.3). The test items were interspersed with these 60 fillers and additional 192 filler items and pseudo-randomized in four list versions. Comprehension questions were constructed for each trial to assure that participants were attentive to the visual presentation. Accuracy rates in this task were on average 94%.

#### Procedure and EEG recording

The same procedures as described for Experiment 1 were used with the exception that trials were presented wordwise (400 ms each and 150 ms ISI). The experimental session consisted of eight blocks of 39 trials each, with brief breaks between blocks.

#### Data analysis

Data analysis also followed the protocol described in Experiment 1. ERPs were time-locked to the onset of the critical noun and averaged per condition and participant. Trials that contained recording artifacts (17%) or registered an incorrect or timed-out response in the comprehension task (6.5%) were discarded prior to averaging. Repeated measures ANOVAs were carried out with the factor CONDITION (Meaning Shift vs. Literal) and the topographical factor ROI/MID (as above) on the mean amplitude value per condition in time windows determined by visual inspection. All analyses were carried out hierarchically and where necessary Huynh and Feldt (1970) corrected.

### Results

**Figure 3:**
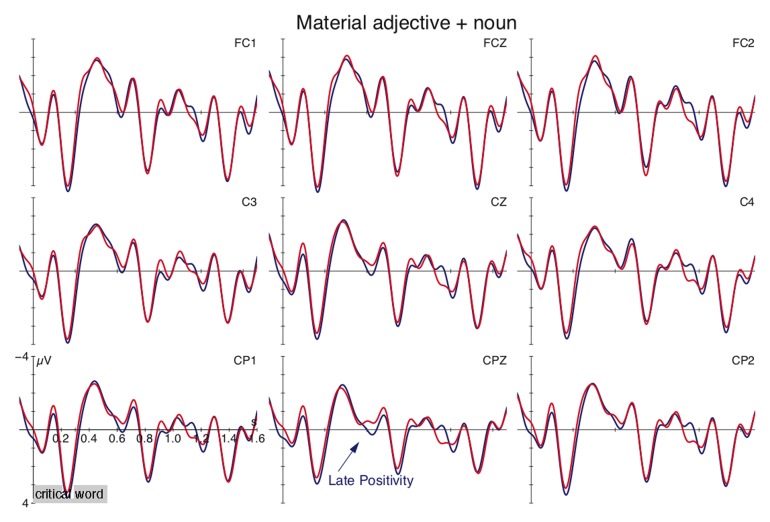
**Grand average ERPs for the adjective-noun combinations, time-locked to the noun (onset at vertical bar) at selected electrode sites for the meaning shift (blue) and control condition (red)**. Negativity is plotted up.

Figure 3 depicts the grand-averages for the combination of material adjectives with nominal expressions for satisfied type presuppositions (e.g., *wooden trunk*) and type mismatches (e.g., *wooden dove*) (onset of noun at vertical bar). The figure shows an enhanced positivity over posterior electrode sites for the shifted use over the literal use between 550-750 ms. This was confirmed by the statistical analysis which registered an interaction of CONDITION × ROI [*F*_(3, 96)_ = 4.23, *p* < 0.03]. Resolution of this interaction by ROI revealed an effect of CONDITION in the left-posterior region [*F*_(1, 32)_ = 4.89, *p* < 0.04]. Additional analyses between 350–500 ms revealed an interaction of CONDITION × ROI [*F*_(3, 96)_ = 4.66, *p* < 0.02] but subsequent resolution registered no significant differences in the individual ROIs. No other time windows yielded a reliable effect. The comparison involving animacy-neutral adjectives (*gray dove* vs. *gray shirt*) registered no differences (see figure in Supplementary material).

### Discussion

Experiment 2 investigated combinatorial operations at the phrasal level by creating a type conflict between a material adjective and an animate entity. The extended interpretation resulted in computational demands reflected in a Late Positive shift relative to a matching adjective-noun combination (cf. Figure 3). Such a Late Positivity has been observed in the container-for-content alternation in Experiment 1 as well as in the *ham sandwich* cases lending support to the proposal that reconceptualization and reference shift engender processing costs. The data further indicate that compositional operations can also be observed at the phrasal level (cf. also Frisson et al., 2011).

The observed effect cannot be viewed as a mere reflex of animacy for the following two reasons. First, the control contrast between animate and inanimate nouns that were combined with animacy-neutral adjectives (*gray dove/shirt*) registered no differences. This suggests that the combinatorial processes are not influenced by animacy information per se. Second, although reanalysis and reconceptualization processes required for *wooden turtle* are triggered by an animacy mismatch between the adjective and the noun, other reanalysis processes did not involve animacy restrictions like the container-for-content alternation in Experiment 1. This indicates that other semantic restrictions influence the underlying processes as well.

## General discussion

The current research wanted to tease apart distinct mechanisms of meaning adjustment. The electrophysiological findings suggest that meaning shifts or metonymy do not represent a uniform phenomenon but are subserved by distinct operations depending on whether the composition leads to referent shift. This is interesting because previous research has on the one hand utilized certain diagnostics such as the copredication test to make claims about the nature of the underlying operations. On the other hand, systematic alternations have by and large been treated alike, as suggested by lists of potential alternations. Crucially, container/content alternations have been classified as bidirectional and as two instances of systematic meaning adjustment. Yet, the current data point toward a more fine-grained dissociation and call for a new typology of meaning shifts. Such a typology should consider the discourse representational consequences of meaning alternations that give rise to either reference shift or meaning selection.

Reference shift represents a process whereby the original denotation of an expression is abandoned in favor of a contextually more appropriate reference. This operation takes place in discourse representation structure where syntax-discourse correspondences result in the introduction of a referent for *the bottle* or *the turtle.* This representation is linked to default lexical information. During combinatorial operations the mismatching information between the predicates and their arguments is encountered leading to resolution mechanisms that draw on lexical information; for example, qualia properties may provide a variable that serves as legitimate referent. The variable represents the intended, unarticulated referent and replaces the original referent. This engenders discourse updating costs. These compositional operations bring about a fundamental reconceptualization of the original referent, and as a consequence, access to the initial interpretation via anaphoric processing is no longer possible.^8^ In other cases, the relationship between the original and the intended meaning is more tightly interconnected, suggesting that one discourse representation makes available both readings simultaneously. This seems to be the case for content expressions like *beer*. This process is termed meaning selection.

The current processing data suggest that reference shift and meaning selection engender distinct processing profiles. Hence, the two alternations involving containers and containables should be treated separately. In content-for-container usage, the two meanings appear to be on a par with each other and equally accessible during utterance processing. The electrophysiological data registered no additional demands during compositional processing when the container reading had to be selected. The container-for-content usage, by contrast, exerted extra computational demands. Taken together with the costs registered for the adjective-noun combinations, the observed positive deflections call for a unified account. This is further corroborated by a Late Positivity effect for *ham sandwich* alternations, a process that can also be ascribed to reference shift from the original denotation to the intended denotation (Schumacher, 2011, 2013).

I suggest that the observed Late Positivities are associated with reconceptualization and modification of discourse representation structure. They thus join other instances of discourse updating triggered by inferences or information structural requirements such as contrastive interpretation (e.g., Burkhardt, 2007; Wang and Schumacher, 2013—cf. Brouwer et al., 2012 for a similar account). This perspective on the Late Positivity is at least partially compatible with previous accounts of semantic P600 effects that attribute mechanisms of conflict resolution to late positive deflections (e.g., Van Herten et al., 2005; Kuperberg et al., 2007; Bornkessel-Schlesewsky and Schlesewsky, 2008). Due to conflicts in composition, semantic reversal anomalies may be remedied or unarticulated meaning constituents may be instantiated. What is new is that seemingly similar conflicts (content-for-container vs. container-for-content) are resolved via distinct processing routes. This calls for a more elaborate involvement of conceptual structure and lexically encoded information.

Animacy has played a prominent role in the discussion of previous Late Positivity effects. The current data—in particular the effects for the container-for-content alternations—add to the view that the underlying mechanism is not specific to animacy but extends to other semantic features such as ontological types as well (Paczynski and Kuperberg, 2011). But could the observed effects reflect implausibility rather than reconceptualization? Even if there is a momentary error detection signal, the pervasiveness of examples of meaning adjustment in everyday life suggests that resolution takes place. The absence of a correlation between offline plausibility ratings and the positivities in the container-for-content study supports this view. Similar null effects were reported for the *ham sandwich* study. These findings indicate that the offline plausibility scores cannot explain the positivities in a satisfactory manner. Disregarding the lacking correlation for a moment, the temporal progression of the neural responses to the container-for-content metonymies as the utterance unfolds could have suggested a two-step process, with the initial positivity reflecting an error detection signal and the second positivity the attempt to rescue the interpretation via meaning adjustment. Such a two-step reconceptualization appears surprising given that other instances of meaning adjustment did not show this pattern (*wooden turtle*, *ham sandwich*), unless one opts for quantitatively different adjustments (cf. Frisson and Frazier, 2005). However, since the initial positivity did not correlate with offline plausibility scores, this explanation forfeits its force.

**Figure 4:**
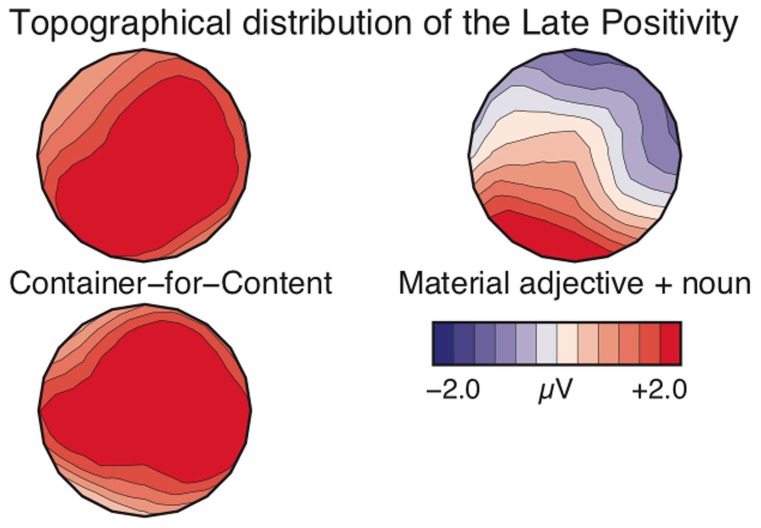
**Topographical distribution of the positivities from container-for-content alternations (left panel) and material adjective + noun combinations (right panel)**. The top row depicts the 550–750 ms window and the bottom row corresponds to the 900–1100 ms window. Frontal electrodes are at top of maps.

Finally, the observed positivities for container-for-content alternations and adjective-noun pairs between 550–750 ms share a maximum distribution over the left posterior region, but differ in their expansion. The positivities for the container-for-content alternations show a broad distribution in both time windows, while the object-denoting animate nouns reveal a more focused effect (see Figure 4). This difference may be due to the nature of the enrichment and possibly the domain of predication (phrase vs. utterance). Furthermore, the similar topographies of the two positivities for container-for-content may indicate that there is an ongoing process rather than two distinct operations.

Two further remarks about the underlying language architecture are warranted. First, the account of computational demands proposed here has a clear focus on the resultant discourse representation. Previous research on compositionality that has targeted metaphor processing has argued for an association of processing cost with suppression of irrelevant information (Gernsbacher and Robertson, 1999; Rubio Fernández, 2007). Given the effects of copredication, however, the findings are not confined to processes associated with lexical representations but show effects at the referential level.

Second, the rationale for adopting a generative lexicon view is that it is not economical for the system to enumerate all meanings as separate lexical entries. This is most apparent from creative uses of object-for-person alternations as in *the ham sandwich.* Instead, lexical entries are flexible and interact with context in important ways such that the lexicon may ultimately provide mechanisms to utilize rich lexical representations. To illustrate this, the current account has been couched within a generative lexicon tradition, because although interpretation is a highly flexible process, the systematicity with which concordant readings are typically generated across hearers calls for access to fine-grained lexical representations or elaborate inferences. Whether this is a Pustejovsky-style system or involves any other formalism cannot be evaluated on the basis of the current design and should be investigated in future research.

It is also noteworthy that no N400 effects were observed in the current experiments. Previous research on compositionality has reported an N400 for complement coercion (Baggio et al., 2010; Kuperberg et al., 2010) and a context-dependent N400 for *ham sandwich* utterances. In the latter case, an N400 emerged when context did not license the referential use, but when a proper scenario was given, no N400 differences were observable between the meaning shift and the control condition (Schumacher, 2011, 2013). Taken together with the absence of an N400 in the three contrasts investigated in the present research, which involved type mismatches, the N400 observed for complement coercion cannot be limited to mismatch detection but appears to reflect early processes of semantic selection.

The present findings complement previous research on meaning shift in vital ways. First, the observation that eye movement measures reveal differences between certain types of metonymies suggests that distinct compositional operations are at work, possibly dissociable along the lines of derivational rule application and lexical selection (cf. for instance producer-for-product shifts and portioning; Frisson and Pickering, 1999; Frisson and Frazier, 2005). If one does not want to commit to such qualitative differences, then a minimum assumption would be that these data point toward quantitatively varying combinatorial costs. The current data add yet another dimension to this by suggesting that lexical processes (whether derivational rules or lexical selection) are to be dissociated from reconceptualization in discourse.

In addition, Frisson and Frazier (2005) reported enhanced processing demands in later eye movement measures for grinding compared to portioning and suggest that this might be an indication of the extra conceptual load associated with grinding. Furthermore, copredication fails in grinding cases (“^*^Mary fed and carved lamb”; cf. Copestake and Briscoe, 1995; Frisson and Frazier, 2005). This provides another testing ground for the present hypothesis. If reconceptualization has to take place during grinding, this should evoke a Late Positivity. Such a finding could also bridge the methodological discrepancies and provide a necessary link between late eye movement measures and the Late Positivity.

## Conclusion

Interpretation is a flexible process that does not exclusively rely on default associations between concepts and expressions but responds to contextual requirements by strengthening appropriate features. Typically, the emerging meanings are not random as suggested by a high degree of accordant interpretations. This gives rise to creative uses of expressions above and beyond the relatively conventional operations that have been investigated in the current research. The present findings indicate that comprehending a referring expression involves setting up a discourse referent. Certain meaning alternations are made available at no further expense by the corresponding discourse representation. In other cases, composition may result in reconceptualization, a process that exerts computational demands. The data thus ask for a new view onto meaning alternations that classifies meaning shifts vis-à-vis their discourse-referential consequences.

### Conflict of interest statement

The author declares that the research was conducted in the absence of any commercial or financial relationships that could be construed as a potential conflict of interest.

## Footnotes

^1^Examples of types are entities and events but also more specific categories such as physical objects, persons, liquids or edibles. The latter are also termed “sorts” in parts of the literature.

^2^Type mismatches are not a necessary prerequisite for meaning shift as exemplified by the referential ambiguity of “The ham sandwich stinks”.

^3^This has no bearing on reference to the original meaning [contra the corresponding ham sandwich example (5.a)], which represents the more critical test case.

^4^Baggio et al. (2010) actually refer to their negativity as a “sustained negativity“ on grounds that it persisted between 300 and 1000 ms.

^5^Stimuli were presented in Dutch verb-final constructions.

^6^From Bloodshot Music: www.bloodshotmusic.com/resources/Johnny%20Drank%20The%20Bottle.mp3.

^7^The metonymic operation underlying the container-for-content alternation can also be found in diachronic developments, such as the English use of dish where the reconceptualization from physical object to edibles has found its way into the lexicon.

^8^It should of course be possible to carry out a subsequent step of reconceptualization in order to direct the attention back to the original denotation. This should however engender processing costs since the interpretation has to be reestablished.
