# Supplementary material for: When combinatorial processing results in reconceptualization: toward a new approach of compositionality
Source: Front Psychol. 2013 Oct 1;4:677. doi: 10.3389/fpsyg.2013.00677 (PMC3787603; doi:10.3389/fpsyg.2013.00677)
Supplement: Supplementary file 1 [file Presentation1.PDF]

Appendix.

A. Stimuli for container-for-content alternations (Experiment 1)
Critical container expressions are in bold-face. The (a)-cases represent the shifted content usages, the (b)-cases represent the container usages.

(1a) Was hat das Neugeborene im Nu getrunken? Es hat **die Babyflasche** im Nu getrunken.
"What did the newborn drink quickly? She drank **the baby bottle** quickly."
(1b) Was hat die Mutter ins Wasserbad gestellt? Sie hat **die Babyflasche** ins Wasserbad gestellt.
"What did the mother put in warm water? She put **the baby bottle** in warm water."

(2a) Was hat Erik im Treppenhaus verschüttet? Er hat **den Putzeimer** im Treppenhaus verschüttet.
"What did Erik spill on the stairs? He spilled **the bucket** on the stairs."
(2b) Was hat Martin auf dem Flohmarkt gekauft? Er hat **den Putzeimer** auf dem Flohmarkt gekauft.
"What did martin buy at the flea market? He bought the bucket at the flea market."

(3a) Was hat die Arzthelferin mit großer Vorsicht injiziert? Sie hat **die Spritze** mit großer Vorsicht injiziert.
"What did the doctor's assistant inject cautiously? She injected **the syringe** cautiously."
(3b) Was hat Paola aus dem Schrank geholt? Sie hat **die Spritze** aus dem Schrank geholt.
"What did Paola get out of the cabinet? She got **the syringe** out of the cabinet."

(4a) Was ist letzten Winter in der Eiseskälte gefroren? Letzten Winter ist **das Planschbecken** in der Eiseskälte gefroren.
"What did freeze last winter in the iciness? Last winter, **the wading pool** froze in the iciness."
(4b) Was ist letzten Sommer in der Gluthitze geplatzt? Letzten Sommer ist **das Planschbecken** in der Gluthitze geplatzt.
"What did burst last summer in the blazing heat? Last summer **the wading pool** burst in the blazing heat."

(5a) Was hat seit 30 Minuten auf dem Herd geköchelt? Seit 30 Minuten hat **der Nudeltopf** auf dem Herd geköchelt.
"What has simmered on the stove for 30 minutes? **The pasta pot** has simmered on the stove for 30 minutes."
(5b) Was hat seit einer Stunde in der Spüle gelegen? Seit einer Stunde hat **der Nudeltopf** in der Spüle gelegen.
"What has been in the sink for an hour? **The pasta pot** has been in the sink for an hour."

(6a) Was hat Markus heimlich verspeist? Er hat **das Nutellaglas** heimlich verspeist.
"What did Markus eat secretly? He eat **the nutella glass** secretly."
(6b) Was hat Anton plötzlich fallen gelassen? Er hat das Nutellaglas plötzlich fallen gelassen.
"What did Anton suddenly drop? He suddenly dropped **the nutella glass**."

(7a) Was hat Fabian in einem Schluck getrunken? Er hat **das Glas** in einem Schluck getrunken.
"What did Fabian drink in one gulp? He drank **the glass** in one gulp."
(7b) Was hat Werner aus Versehen verloren? Er hat **das Glas** aus Versehen verloren.
"What did Werner loose accidentally? He lost **the glass** accidentally."

(8a) Was hat Heinz hastig getrunken? Er hat **den Becher** hastig getrunken.
"What did Heinz drink hastily? He drank **the goblet** hastily."
(8b) Was hat Rolf wie seinen Augapfel gehütet? Er hat den Becher wie seinen Augapfel gehütet.
"What did Rolf guard jealously? He guarded **the goblet** jealously."

(9a) Was hat Holger in Sekundenschnelle getrunken? Er hat **die Wasserflasche** in Sekundenschnelle getrunken.
"What did Holger drink in a matter of seconds? He drank **the water bottle** in a matter of seconds."
(9b) Was hat Andreas in seiner Hand gehalten? Er hat **die Wasserflasche** in seiner Hand gehalten.
"What did Andreas hold in his hand? He held **the water bottle** in his hand."

(10a) Was hat Tony eilig verschlungen? Er hat das Joghurtglas eilig verschlungen.
"What did Tony devour quickly? He devoured **the yoghurt jar** quickly."
(10b) Was hat Monika in den Container geworfen? Sie hat das Joghurtglas in den Container geworfen.
"What did Monika throw in the garbage container? She threw **the yoghurt jar** in the garbage container."

(11a) Was hat seit drei Stunden auf dem Lagerfeuer gekocht? Seit drei Stunden hat **der Kupferkessel** auf dem Lagerfeuer gekocht.
"What has boiled on the campfire for three hours? **The copper kettle** has boiled on the campfire for three hours."
(11b) Was hat seit einer Stunde im Wasser gelegen? Seit einer Stunde hat der Kupferkessel im Wasser gelegen.
"What has been in the water for an hour? **The copper kettle** has been in the water for an hour."

(12a) Was hat seit einer Stunde auf dem Herd gekocht? Seit einer Stunde hat **der Teekessel** auf dem Herd gekocht.
"What has boiled on the oven for an hour? **The teakettle** has boiled on the oven for an hour."
(12b) Was ist vorhin vom Herd gerutscht? Vorhin ist **der Teekessel** vom Herd gerutscht.
"What has fallen off the oven a little while ago? **The teakettle** has fallen off the oven a little while ago."

(13a) Was hat der Vielfraß als Nachtisch vertilgt? Er hat **die Chipstüte** zum Nachtisch vertilgt.
"What did the gourmand devour for dessert? He devoured **the bag of chips** for dessert."
(13b) Was hat Maria auf den Tisch gelegt? Sie hat **die Chipstüte** auf den Tisch gelegt.
"What did Maria place on the table? She place **the bag of chips** on the table."

(14a) Was hat der Randalierer bei der Demo versprüht? Er hat **den Feuerlöscher** bei der Demo versprüht.
"What did the troublemaker spray during the demonstration. He sprayed **the fire extinguisher** during the demonstration."
(14b) Was hat der Feuerwehrmann routinemäßig überprüft? Er hat **den Feuerlöscher** routinemäßig überprüft.
"What die the fire fighter inspect routinely? He inspected **the fire extinguisher** routinely."

(15a) Was hat Silvias Tochter innerhalb wenigen Minuten versprüht? Sie hat **den Parfümflakon** innerhalb wenigen Minuten versprüht.
“What did Silvia’s daughter use up within a few minutes? She used up **the perfume bottle** within a few minutes.”
(15b) Was hat der Teufelsbraten aus Langeweile versteckt? Er hat **den Parfümflakon** aus Langeweile versteckt.
“What did the little devil hide due to boredom? He hid **the perfume bottle** due to boredom.”

(16a) Was hat das Kind heimlich verspeist? Es hat **die Pralinenschachtel** heimlich verspeist.
“What did the child consume secretly? He consumed **the box of chocolates** secretely.”
(16b) Was hat Jonas liebevoll verpackt? Er hat **die Pralinenschachtel** liebevoll verpackt.
“What did Jonas wrap attentively? He wrapped **the box of chocolates** attentively.”

(17a) Was hat der Sportler nach der Etappe getrunken? Er hat **die Fahrradflasche** nach der Etappe getrunken.
“What did the sportsman drink after the stage? He drank **the cycling bottle** after the stage.”
(17b) Was hat der Freizeitsportler in seinen Rucksack gepackt? Er hat **die Fahrradflasche** in seinen Rucksack gepackt.
“What die the recreational sportsman put in his backpack? He put **the cycling bottle** in his backpack.”

(18a) Was hat seit zwei Stunden auf dem Ofen gekocht? Seit zwei Stunden hat **der Topf** auf dem Ofen gekocht.
“What has seethed on the stove for two hours? **The kettle** has seethed on the stove for two hours.”
(18b) Was hat seit einer Stunde in der Spülmaschine gelegen? Seit einer Stunde hat **der Topf** in der Spülmaschine gelegen.
“What has been in the dishwasher for an hour? **The kettle** has been in the dishwasher for an hour.”

(19a) Was hat Peter flugs vertilgt? Er hat **den Eisbecher** flugs vertilgt.
“What did Peter devour quickly? He devoured **the sundae dish** quickly.”
(19b) Was hat der Eisverkäufer auf den Tresen gestellt? Er hat **den Eisbecher** auf den Tresen gestellt.
“What did the ice-cream seller place on the counter? He place **the sundae dish** on the counter.”

(20a) Was ist im Garten als es regnete übergelaufen? Als es regnete ist **das Fischbecken** im Garten übergelaufen.
“What did overflow in the backyard when it rained? When it rained, **the fishpond** overflew in the backyard.”
(20b) Was hat der Gärtner fachmännisch abgedichtet? Er hat **das Fischbecken** fachmännisch abgedichtet.
“What did the gardener seal competently? He sealed **the fishpond** competently.”

(21a) Was hat der unachtsame Kranfahrer aus Versehen verschüttet? Er hat **den Schmelztiegel** aus Versehen verschüttet.
“What did the careless crane operator spill inadvertently? He spilled **the smelter** inadvertently.”

(21b) Was hat der Kran mit Leichtigkeit angehoben? Er hat **den Schmelztiegel** mit Leichtigkeit angehoben.
"What did the crane lift effortlessly? The crane lifted **the smelter** effortlessly."

(22a) Was hat der Maler aus Achtlosigkeit verschüttet? Er hat **den Farbeimer** aus Achtlosigkeit verschüttet.
"What did the painter spill thoughtlessly? He spilled **the paint bucket** thoughtlessly."
(22b) Was hat Gerd in Windeseile versiegelt? Er hat **den Farbeimer** in Windeseile versiegelt.
"What did Gerd seal in next to no time? He sealed **the paint bucket** in next to no time."

(23a) Was ist seit dem Umweltskandal chronisch übersäuert? Seit dem Umweltskandal ist **das Becken** chronisch übersäuert.
"What has suffered from chronic acidification since the environmental scandal? Since the environmental scandal, **the basin** has suffered from chronic acidification."
(23b) Was ist letzten Winter mehrmals zugefroren? Letzten Winter ist **das Becken** mehrmals zugefroren.
"What has frozen over several times last winter? **The basin** has frozen over several times last winter."

(24a) Was hat der Bayer genussvoll hinuntergekippt? Er hat **den Bierkrug** genussvoll hinuntergekippt.
"What did the Bavarian knock back with obvious enjoyment? He knocked back **the stein** with obvious enjoyment."
(24b) Was hat die Angestellte auf dem Tablett balanciert? Sie hat **den Bierkrug** auf dem Tablett balanciert.
"What did the employee balance on the tray? She balanced **the stein** on the tray."

(25a) Was hat der Zoomitarbeiter ungewollt überdüngt? Er hat **das Aquarium** ungewollt überdüngt.
"What did the zoo employee over-fertilize unintentionally? He over-fertilized **the fish tank** unintentionally."
(25b) Was hat Tom mit Eifer geputzt? Er hat **das Aquarium** mit Eifer geputzt.
"What did Tom clean diligently? He cleaned **the fish tank** diligently."

(26a) Was hat Felix aus Versehen sauer werden lassen? Er hat **die Milchpackung** aus Versehen sauer werden lassen.
"What did Felix cause to get sour by accident? He cause **the milk carton** to get sour by accident."
(26b) Was hat Thomas nach dem Frühstück weggeschmissen? Er hat **die Milchpackung** nach dem Frühstück weggeschmissen.
What did Thomas throw away after breakfast? He through away **the milk carton** after breakfast."

(27a) Was ist vor Monaten durch Algen trübe geworden? Vor Monaten ist **der Swimmingpool** durch Algen trübe geworden.
"What has turned cloudy from algae a few months ago? A few months ago, **the swimming pool** has turned cloudy from algae."
(27b) Was ist letzten Sommer fertig gebaut worden? Letzten Sommer ist **der Swimmingpool** fertig gebaut worden.
"What has been completed last summer? Last summer, **the swimming pool** has been completed."

(28a) Was hat das Abwasser seit Jahren vergiftet? Es hat **den Brunnen** seit Jahren vergiftet.
"What has been contaminated by the sewage for years? For years, **the well** has been contaminated by the sewage."
(28b) Was hat der Bauarbeiter plangemäß zugemauert? Er hat **den Brunnen** plangemäß zugemauert.
"What did the construction worker brick up on schedule? He bricked up **the well** on schedule."

(29a) Was hat der Alchemist furchtlos eingenommen? Er hat **die Giftphiole** furchtlos eingenommen.
"What did the alchemist take intrepidly? He took **the poison phial** intrepidly."
(29b) Was hat der Schwarzmagier in der Truhe versteckt? Er hat **die Giftphiole** in der Truhe versteckt.
"What did the black magician hide in the trunk? He hid **the poison phial** in the trunk."

(30a) Was hat die Chirurgin schon vor Dienstbeginn getrunken? Sie hat **die Kaffeekanne** vor Dienstbeginn getrunken.
"What has the surgeon already had before work? She has had **the coffee pot** before work."
(30b) Was hat der Hotelangestellte auf den Tisch gestellt? Er hat **die Kaffeekanne** auf den Tisch gestellt.
"What did the hotel employee place on the table? She placed **the coffee pot** on the table."

(31a) Was hat Ute im Kaufrausch verprasst? Sie hat **das Sparschwein** im Kaufrausch verprasst.
"What did Ute squander in a buying spree? She squander **the piggy bank** in a buying spree."
(31b) Was hat Johann mit dem Hammer zertrümmert? Er hat **das Sparschwein** mit dem Hammer zertrümmert.
"What did Johann smash with a hammer? He smashed **the piggy bank** with a hammer."

(32a) Was hat der Alkoholiker gierig hinuntergekippt? Er hat **die Bierflasche** gierig hinuntergekippt.
"What did the alcohol addict knock back greedily? He knocked back **the bottle of beer** greedily."
(32b) Was hat der Unruhestifter gegen das Fenster geworfen? Er hat **die Bierflasche** gegen das Fenster geworfen.
"What did the agitator throw at the window? He threw **the bottle of beer** at the window."

(33a) Was hat Alfred zügig hinuntergeschlungen? Er hat **den Puddingbecher** zügig hinuntergeschlungen.
"What did Alfred swallow rapidly? He swallowed **the custard cup** rapidly."
(33b) Was hat Fred an Maria weitergereicht? Er hat **den Puddingbecher** an Maria weitergereicht.
"What did Fred pass on to Maria? He passed **the custard cup** on to Maria."

(34a) Was hat der Schluckspecht während der Arbeitszeit getrunken? Er hat **die Rotweinflasche** während der Arbeitszeit getrunken.
"What did the boozer drink during his working hours? He drank **the red wine bottle** during his working hours."
(34b) Was hat der Trunkenbold im Einkaufswagen vor sich hergeschoben? Er hat **die Rotweinflasche** im Einkaufswagen vor sich hergeschoben.

“What did the drunkard push around in the shopping cart? He pushed around **the red wine bottle** in the shopping cart.”

(35a) Was hat der Delinquent heimlich in seinen Mund geträufelt? Er hat **die Glasampulle** heimlich in seinen Mund geträufelt.
“What did the delinquent dribble in his mouth? He dribbled **the glass ampulla** in his mouth.”
(35b) Was hat der Apotheker im Schrank verstaut? Er hat **die Glasampulle** im Schrank verstaut.
“What did the pharmacist secure in the cupboard? He secured **the glass ampulla** in the cupbouard.”

(36a) Was hat der Geistliche beim Abendmahl ganz alleine getrunken? Er hat **den Kelch** ganz alleine getrunken.
“What did the clergyman drink by himself during Communion? He drank **the goblet** by himself during Communion.
(36b) Was hat der Ministrant nach dem Gottesdienst gereinigt? Er hat **den Kelch** nach dem Gottesdienst gereinigt.
“What did the altar boy clean after church? He cleaned **the goblet** after church.”

(37a) Was hat der Partygast in kürzester Zeit getrunken? Er hat **den Sangriaeimer** in kürzester Zeit getrunken.
“What did the guest drink in a very short time? He drank **the bucket of sangria** in a very short time.”
(37b) Was hat Ricardo am Strand umgestoßen? Er hat **den Sangriaeimer** am Strand umgestoßen.
“What did Ricardo knowck over at the beach? He knocked over **the bucket of sangria** at the beach.”

(38a) Was hat der Gärtner über dem Blumenbeet vergossen? Er hat **die Regentonne** über dem Blumenbeet vergossen.
“What did the gardener spill over the flower bed? He spilled **the rain barrel** over the flower bed.”
(38b) Was hat der Hausbesitzer in den Garten gestellt? Er hat **die Regentonne** in den Garten gestellt.
“What did the house owner put in the backyard? He put **the rain barrel** in the backyard.”

(39a) Was hat der Hausmeister bereits zum Frühstück getrunken? Er hat **die Whiskyflasche** zum Frühstück getrunken.
“What has the janitor drunk for breakfast already? He has drunk **the whiskey bottle** for breakfast.”
(39b) Was hat der Sicherheitsmann ins Altglas geworfen? Er hat **die Whiskyflasche** ins Altglas geworfen.
“What did the security guy toss in the glass waste? He tossed **the whiskey bottle** in the glass waste.”

(40a) Was hat der Chemiker zum Kochen gebracht? Er hat **den Glaskolben** zum Kochen gebracht.
“What did the chemist bring to a boil? He brought **the glass flask** to a boil.”
(40b) Was hat der Laborant mit Wasser gereinigt? Er hat **den Glaskolben** mit Wasser gereinigt.
“What did the lab assistant cleanse with water? He cleansed **the glass flask** with water.”

B. Stimuli for content-for-container alternations (Experiment 1)
Critical content expressions are in bold-face. The (a)-cases represent the shifted container interpretation, the (b)-cases represent the content interpretation.

(1a) Was hat Asterix an seinem Gürtel festgeschnallt? Er hat **den Zaubertrank** an seinem Gürtel festgeschnallt.
"What did Asterix fasten to his belt? He fastened **the magic potion** to his belt."
(1b) Was hat Miraculix vor dem Eintreffen der Römer gebraut? Er hat **den Zaubertrank** vor dem Eintreffen der Römer gebraut.
"What did Miraculix brew before the arrival of the Romans? He brewed **magic potion** before the arrival of the Romans."

(2a) Was hat Eugen in den Kühlschrank gestellt? Er hat **den Orangensaft** in den Kühlschrank gestellt.
"What did Eugen put in the fridge? He put **the orange juice** in the fridge."
(2b) Was hat Thomas in das Glas gefüllt? Er hat **den Orangensaft** in das Glas gefüllt.
"What did Thomas pour in the glass? He poured **orange juice** in the glass."

(3a) Was hat Nina mit dem Feuerzeug geöffnet? Sie hat **das Pils** mit dem Feuerzeug geöffnet.
"What did Nina open with the lighter? She opened **the Pilsener** with the lighter."
(3b) Was hat Elena nach der Arbeit getrunken? Sie hat **das Pils** nach der Arbeit getrunken.
"What did Elena drink after work? She drank **Pilsener** after work."

(4a) Was hat Nicole aus Unachtsamkeit umgeworfen? Sie hat **den Nagellack** aus Unachtsamkeit umgeworfen.
"What did Nicole knock over due to inattentiveness? She knocked over **the nail polish** due to inattentiveness."
(4b) Was hat Bianca auf ihre Hose geschmiert? Sie hat **den Nagellack** auf ihre Hose geschmiert.
"What did Bianca smear on her pants? She smeared **nail polish** on her pants."

(5a) Was hat Klaus in den Vorratsraum gestellt? Er hat **die Limonade** in den Vorratsraum gestellt.
"What did Klaus put in the storage room? He put **the lemonade** in the storage room."
(5b) Was hat Martin aus der Flasche getrunken? Er hat **die Limonade** aus der Flasche getrunken.
"What did marktin drink out of the bottle? He drank **lemonade** out of the bottle."

(6a) Was hat der Eingeborene auf seinem Kopf balanciert? Er hat **das Brunnenwasser** auf seinem Kopf balanciert.
"What did the indigene balance on his head? He balanced **the well water** on his head."
(6b) Was hat der Landwirt in den Kanister gefüllt? Er hat **das Brunnenwasser** in den Kanister gefüllt.
"What did the farmer fill in the can? He filled **well water** in the can."

(7a) Was hat der Ober auf den Tisch gestellt? Er hat **den Champagner** auf den Tisch gestellt.
"What did the waiter place on the table? He placed **the champagne** on the table."

(7b) Was hat der Gourmet im Restaurant genossen? Er hat **den Champagner** im Restaurant genossen.
"What did the gourmet enjoy at the restaurant? He enjoyed **champagne** at the restaurant."

(8a) Was hat Miriam in den Einkaufskorb fallen gelassen? Sie hat **das Haarshampoo** in den Einkaufskorb fallen gelassen.
"What did Miriam drop in the shopping basket? She dropped **the shampoo** in the shopping basket."
(8b) Was hat Gisela aus Versehen ins Auge bekommen? Sie hat **das Haarshampoo** aus Versehen ins Auge bekommen.
"What did Gisela accidentally get in her eyes? She accidentally got **shampoo** in her eyes."

(9a) Was hat Andreas beim Kochen umgeworfen? Er hat **das Öl** beim Kochen umgeworfen.
"What did Andreas knock over while cooking? He knocked over **the oil** while cooking."
(9b) Was hat Robert am Auto nachgefüllt? Er hat **das Öl** am Auto nachgefüllt.
"What did Robert refill his car with? He refilled his car with **oil**."

(10a) Was hat der Dieb in seiner Jackentasche versteckt? Er hat **den Whisky** in seiner Jackentasche versteckt.
"What did the thief hide in his pocket? He hid **the whiskey** in his pocket."
(10b) Was hat der Alkoholiker in rauen Mengen getrunken? Er hat **den Whisky** in rauen Mengen getrunken.
"What did the alcohol addict drink in great quantities? He drank **whiskey** in great quantities."

(11a) Was hat Helmut im Einkaufswagen vergessen? Er hat **die Cola** im Einkaufswagen vergessen.
"What did Helmut leave in the shopping cart? He left **the coke** in the shopping cart."
(11b) Was hat Jens gestern verschüttet? Er hat **die Cola** gestern verschüttet.
"What did Jens spill yesterday? Yesterday, he spilled **coke**."

(12a) Was hat die Köchin aus dem Schrank geholt? Sie hat **den Essig** aus dem Schrank geholt.
"What did the chef fetch from the cupboard? She fetched **the vinegar** from the cupboard."
(12b) Was hat Julia über den Salat geleert? Sie hat **den Essig** über den Salat geleert.
"What did Julie pour on the salad? She poured **vinegar** on the salad."

(13a) Was hat der Morgenmuffel aus Versehen umgeworfen? Er hat **das Salz** aus Versehen umgeworfen.
"What did the guy (who is grumpy in the mornings) knock over by accident? He knocked over **the salt** by accident."
(13b) Was hat der Küchenchef über das Gemüse gestreut? Er hat **das Salz** über das Gemüse gestreut.
"What did the chef sprinkle on the vegetables? He sprinkled **salt** on the vegetables."

(14a) Was hat die Bedienung auf den Tisch gestellt? Sie hat **den Pfeffer** auf den Tisch gestellt.
"What did the waitress put on the table? She put **the pepper** on the table."
(14b) Was hat die Chefköchin im Mörser zerkleinert? Sie hat **den Pfeffer** im Mörser zerkleinert.
"What did the chef grind in the mortar? She ground **pepper** in the mortar."

(15a) Was hat der Bauer nach dem Melken umgekippt? Er hat **die Milch** nach dem Melken umgekippt.
"What did the farmer tip over after milking? He tipped over **the milk** after milking."
(15b) Was hat Inge im Stehen getrunken? Sie hat **die Milch** im Stehen getrunken.
"What did Inge drink while standing? She drank **milk** while standing."

(16a) Was hat die Mutter im Wasserbad stehen? Sie hat **den Babybrei** im Wasserbad stehen.
"What did the mother place in the bain-marie? She placed **the baby mash** in the bain-marie.
(16b) Was hat das Kleinkind auf das Lätzchen gekleckert? Es hat **den Babybrei** auf das Lätzchen gekleckert.
"What did the infant spill on the bib? He spilled **baby mash** on the bib."

(17a) Was ist heute Morgen lautstark auf den Boden gefallen? Heute Morgen ist **das Parfüm** lautstark auf den Boden gefallen.
"What has dropped on the floor noisily this morning? This morning, **the perfume** has dropped on the floor noisily."
(17b) Was hat Sebastian in den Abfluss gegossen? Er hat **das Parfüm** in den Abfluss gegossen.
"What did Sebastian pour down the sink? He poured **perfume** down the sink."

(18a) Was ist heute Morgen aus dem Gewürzregal gerutscht? Heute Morgen ist **der Koriander** aus dem Gewürzregal gerutscht.
"What has skittered off the spice shelf this moring? This moring, **the coriander** has skittered off the spice shelf."
(18b) Was hat gestern die Soße verfeinert? Gestern hat **der Koriander** die Soße verfeinert.
"What did refine the gravy yesterday? **Coriander** refined the gravy yesterday."

(19a) Was hat der Braumeister aus dem Lager gerollt? Er hat **das Bier** aus dem Lager gerollt.
"What did the master brewer roll out off the warehouse? He rolled **the beer** out off the warehouse."
(19b) Was hat der Wirt in ein Glas gefüllt? Er hat **das Bier** in ein Glas gefüllt.
"What die the innkeeper pour in a glass? He poured **beer** in a glass."

(20a) Was hat Alexandra vom Herd genommen? Sie hat **das Teewasser** vom Herd genommen.
"What did Alexandra take off the stove? She took **the tea water** off the stove."
(20b) Was hat Simone in die Tasse gegossen? Sie hat **das Teewasser** in die Tasse gegossen.
"What did Simone pour in the cup? She poured **tea water** in the cup."

(21a) Was hat die Aushilfe ins Regal geräumt? Sie hat **die Flüssigseife** ins Regal geräumt.
"What did the temporary worker place on the shelves? She placed **the liquid soap** on the shelves."
(21b) Was hat Nina in ein Fläschchen umgefüllt? Sie hat **die Flüssigseife** in ein Fläschchen umgefüllt.
"What did Nina transfer into a flask? She transfer **liquid soap** into a flask."

(22a) Was hat der Lagerarbeiter auf Paletten gestapelt? Er hat **das Waschmittel** auf Paletten gestapelt.
"What did the storeman palletize? He palletized **the laundry detergent**."
(22b) Was hat Tina sorgfältig abgemessen? Sie hat **das Waschmittel** sorgfältig abgemessen.
"What did Tina gauge meticulously? She gauged **laundry detergent** meticulously."

(23a) Was hat der Packer in Getränkekisten sortiert? Er hat **das Mineralwasser** in Getränkekisten sortiert.
"What did the packer arrange into crates? He arranged **the mineral water** into crates."
(23b) Was hat Guido an die Pflanzen gekippt? Er hat **das Mineralwasser** an die Pflanzen gekippt.
"What did Guido dump onto the plants? He dumped **mineral water** onto the plants."

(24a) Was hat der Koch mit dem Ellenbogen umgestoßen? Er hat **die Bratensoße** mit dem Ellenbogen umgestoßen.
"What did the chef knock over with his elbow? He knocked over **the gravy** with his elbow."
(24b) Was hat Peter nur ungern weggeschüttet? Er hat **die Bratensoße** nur ungern weggeschüttet.
"What did Peter tip away reluctantly? He tipped away **gravy** reluctantly."

(25a) Was hat der Sommelier aus dem Keller geholt? Er hat **den Wein** aus dem Keller geholt.
"What did the sommelier get in the basement? He got **the wine** in the basement."
(25b) Was hat Alfred auf der Messe verkostet? Er hat **den Wein** auf der Messe verkostet.
"What did Alfred taste at the fair? He tasted **wine** at the fair."

(26a) Was hat Bernd ins Regal gestellt? Er hat **die Cornflakes** ins Regal gestellt.
"What did Bernd put on the shelves? He put **the cornflakes** on the shelves."
(26b) Was hat Katrin in die Schüssel geschüttet? Sie hat **die Cornflakes** in die Schüssel geschüttet.
"What did Katrin pur in the bowl? She poured **cornflakes** in the bowl."

(27a) Was ist nach dem Einkauf im Auto geplatzt? Nach dem Einkauf ist **die Sprühsahne** im Auto geplatzt.
"What did burst in the car after shopping? After shopping, **the aerosol cream** burst in the car."
(27b) Was hat Christine genussvoll gelöffelt? Sie hat **die Sprühsahne** genussvoll gelöffelt.
"What did Christine spoon with pleasure? She spooned **aerosol cream** with pleasure."

(28a) Was hat Lisa am Beckenrand umgestoßen? Sie hat **die Sonnencreme** am Beckenrand umgestoßen.
"What did Lisa knock over near the edge of the pool? She knocked over **the suntan lotion** near the edge of the pool."
(28b) Was hat Tim auf Susis Rücken verrieben? Er hat **die Sonnencreme** auf Susis Rücken verrieben.
"What did Tim apply to Suzy's back? He applied **suntan lotion** to Suzy's back."

(29a) Was hat der Barbier vom Tablett geschubbst? Er hat **das Rasierwasser** vom Tablett geschubbst.
"What did the barber shove off the tray? He shoved **the aftershave** off the tray."
(29b) Was hat heute Morgen auf Davids Haut gebrannt? Heute Morgen hat **das Rasierwasser** auf Davids Haut gebrannt.
"What has burned on David's skin this morning? This morning, **the aftershave** has burned on David's skin."

(30a) Was hat die Masseurin neben die Liege gestellt? Sie hat **das Massageöl** neben die Liege gestellt.

“What did the masseuse put next to the couch? She put **the lotion** next to the couch.”
(30b) Was hat Tanja in ihre Handflächen geträufelt? Sie hat **das Massageöl** in ihre Handflächen geträufelt.
“What did Tanja drizzle on her palms? She drizzled **lotion** on her palms.”

(31a) Was hat der Chemielaborant in den Vorratsraum gestellt? Er hat **die Säure** in den Vorratsraum gestellt.
“What did the laboratory assistant place in the store room? He placed **the acid** in the store room.”
(31b) Was hat der Chemielehrer nach dem Unterricht entsorgt? Er hat **die Säure** nach dem Unterricht entsorgt.
“What did the chemistry teacher dispose of after class? He disposed of **acid** after class.”

(32a) Was hat der Barmann aus dem Regal genommen? Er hat **den Gin** aus dem Regal genommen.
“What did the barkeeper take from the shelves? He took **the gin** from the shelves.”
(32b) Was hat Michael ins Glas gegossen? Er hat **den Gin** ins Glas gegossen.
“What did Michael pour into the glass? He poured **gin** into the glass.”

(33a) Was hat die Empfangsdame auf das Tablett gestellt? Sie hat **den Sekt** auf das Tablett gestellt.
“What did the receptionist place on the tray? She placed **the sparkling wine** on the tray.”
(33b) Was hat Andrea mit Hibiskusblüte getrunken? Sie hat **den Sekt** mit Hibiskusblüte getrunken.
“What did Andrea drink with hibiscus blossom? She drank **sparkling wine** with hibiscus blossom.”

(34a) Was hat Natalie auf den Herd gestellt? Sie hat **die Suppe** auf den Herd gestellt.
“What did Natalie put on the stove? She put **the soup** on the stove.”
(34b) Was hat Tom mit Salz abgeschmeckt? Er hat **die Suppe** mit Salz abgeschmeckt.
“What did Tom salt to taste? He salted to taste **the soup**.”

(35a) Was hat der Abteilungsleiter ins Regal eingeräumt? Er hat **den Sambuca** ins Regal eingeräumt.
“What did the department manager move to the shelves? He moved **the sambuca** to the shelves.”
(35b) Was hat der Barkeeper im Glas entflammt? Er hat **den Sambuca** im Glas entflammt.
“What did the barkeeper inflame in the glass? He inflamed **sambuca** in the glass.”

(36a) Was hat der Zahnarzt in den Schrank geräumt? Er hat **die Mundspülung** in den Schrank geräumt.
“What did the dentist move into the cupboard? He moved **the mouthwash** into the cupboard.”
(36b) Was hat Marianne ins Waschbecken gespuckt? Sie hat **die Mundspülung** ins Waschbecken gespuckt.
“What did Marianne spit in the sink? She spat **mouthwash** in the sink.”

(37a) Was hat Dorothé in die Kühlschranktür gestellt? Sie hat **das Ketchup** in die Kühlschranktür gestellt.
“What did Dorothé store in the door of the fridge? She stored **the ketchup** in the door of the fridge.”

(37b) Was hat Mareike von den Pommes geleckt? Sie hat **das Ketchup** von den Pommes geleckt.
"What did Mareike lick off the French Fries? She licked **ketchup** off the French Fries."

(38a) Was hat der Imbissbetreiber auf die Theke gestellt? Er hat **die Mayonnaise** auf die Theke gestellt.
"What did the owner of the snackbar put on the counter? He put **the mayonnaise** on the counter."
(38b) Was hat Kevin auf den Burger gestrichen? Er hat **die Mayonnaise** auf den Burger gestrichen.
"What did Kevin spread on the burger? He spread **mayonnaise** on the burger."

(39a) Was hat der Winzer ins Weinregal gelegt? Er hat **den Weißburgunder** ins Regal gelegt.
"What did the winegrower place on the wine shelves? He places **the Pinot Blanc** on the wine shelves."
(39b) Was hat der Pfarrer gern getrunken? Er hat **den Weißburgunder** gern getrunken.
"What did the pastor like to drink? He liked to drink **Pinot Blanc**."

(40a) Was hat der Apotheker in den Arzneischrank gestellt? Er hat **den Hustensaft** in den Arzneischrank gestellt.
"What did the pharmacist store in the medicine cabinet? He stored **the cough syrup** in the medicine cabinet."
(40b) Was hat das Kind ungern eingenommen? Es hat **den Hustensaft** ungern eingenommen.
"What did the child take reluctantly? She took **cough syrup** reluctantly."

C. Stimuli for adjective-noun combinations (Experiment 2)

Critical adjective-noun pairs for Experiment 2. All utterances were of the sort illustrated in Table 3. The first noun is part of the shifted interpretations, the second noun represents the control condition.

(1) die hölzerne Taube / Truhe
"the wooden dove / trunk"

(2) der gusseiserne Löwe / Löffel
"the cast-iron lion / spoon"

(3) die seidene Giraffe / Girlande
"the silk giraffe / garland"

(4) der gläserne Sperber / Leuchter
"the glas sparrowhawk / candelabrum"

(5) der blecherne Eisbär / Eimer
"the tinny polar bear / bucket"

(6) das porzellanene Krokodil / Kännchen
"the china crocodile / little-pot"

(7) die kristallene Meise / Vase
“the crystalline titmouse / vase”

(8) der diamantene Kiebitz / Kasten
“the diamond lapwing / case”

(9) der gläserne Tapir / Kugel
“the glas tapir / bowl”

(10) der metallene Spatz / Stab
“the metallic sparrow / bar”

(11) der papierne Bär / Block
“the paper bear / pad”

(12) die lederne Eule / Hose
“the leathern owl / pants”

(13) der gusseiserne Papagei / Apparat
“the cast-iron parrot / apparatus”

(14) die tönerne Fliege / Fliese
“the fictile fly / tile”

(15) der bernsteinerne Spatz / Schatz
“the amber sparrow / treasure”

(16) der bleierne Adler / Anker
“the lead eagle / anchor”

(17) die filzene Biene / Mütze
“the felt bee / bonnet”

(18) die metallene Ameise / Antenne
“the metallic ant / antenna”

(19) der eiserne Biber / Becher
“the iron beaver / goblet”

(20) der lederne Vogel / Vorhang
“the leathern bird / curtain”

(21) die blecherne Tarantel / Schatulle
“the tinny tarantula / jewel case”

(22) die tönerne Schabe / Scherbe
“the fictile cockroach / shard”

(23) das hölzerne Rebhuhn / Regal
“the wooden partridge / shelf”

(24) das schieferne Schwein / Schild
"the schist pig / plate"

(25) die steinerne Wachtel / Wanne
"the stone quail / tub"

(26) die kristallene Schnecke / Schale
"the crystalline snail / dish"

(27) die papierne Schlange / Lasche
"the paper snake / flap"

(28) der eiserne Steinbock / Spaten
"the iron Capricorn / spade"

(29) die styroporne Drossel / Dämmung
"the styrofoam thrush / insulation"

(30) die porzellanene Katze / Kanne
"the china cat / pitcher"
