# Supplementary material for: When combinatorial processing results in reconceptualization: toward a new approach of compositionality
Source: Front Psychol. 2013 Oct 1;4:677. doi: 10.3389/fpsyg.2013.00677 (PMC3787603; doi:10.3389/fpsyg.2013.00677)

Figure A: Grand average ERPs for adjective-noun combinations requiring no reference shift on the animate noun. ERPs are time-licked to the noun (onset at vertical bar).

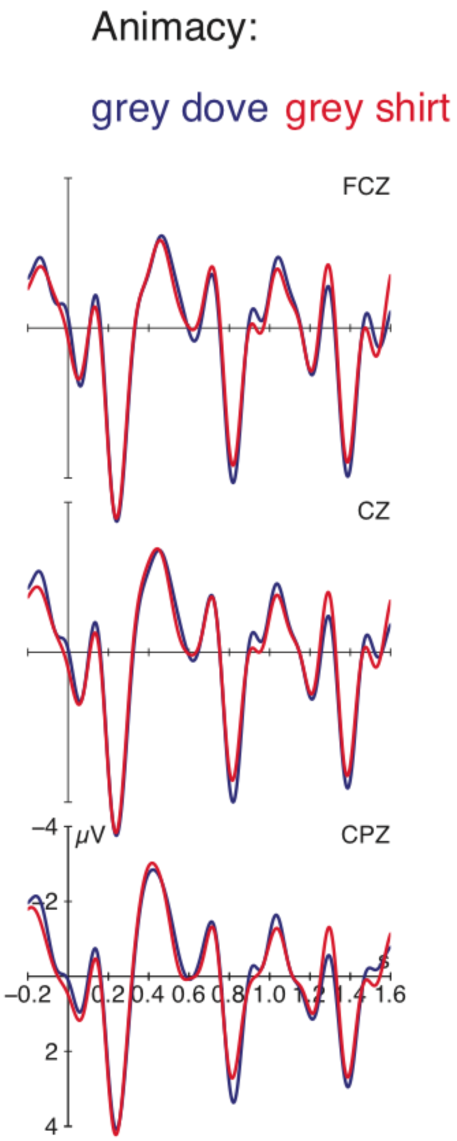

Supplement: Supplementary file 2 [file Presentation2.PDF]
